# A deep learning framework to discern and count microscopic nematode eggs

**DOI:** 10.1038/s41598-018-27272-w

**Published:** 2018-06-14

**Authors:** Adedotun Akintayo, Gregory L. Tylka, Asheesh K. Singh, Baskar Ganapathysubramanian, Arti Singh, Soumik Sarkar

**Affiliations:** 1 Iowa State University, Mechanical Engineering Department, Ames, 50011 USA; 2 Iowa State University, Plant Pathology and Microbiology Department, Ames, 50011 USA; 3 Iowa State University, Agronomy Department, Ames, 50011 USA

## Abstract

In order to identify and control the menace of destructive pests via microscopic image-based identification state-of-the art deep learning architecture is demonstrated on the parasitic worm, the soybean cyst nematode (SCN), *Heterodera glycines*. Soybean yield loss is negatively correlated with the density of SCN eggs that are present in the soil. While there has been progress in automating extraction of egg-filled cysts and eggs from soil samples counting SCN eggs obtained from soil samples using computer vision techniques has proven to be an extremely difficult challenge. Here we show that a deep learning architecture developed for *rare object identification in clutter*-*filled images* can identify and count the SCN eggs. The architecture is trained with expert-labeled data to effectively build a machine learning model for quantifying SCN eggs via microscopic image analysis. We show dramatic improvements in the quantification time of eggs while maintaining human-level accuracy and avoiding inter-rater and intra-rater variabilities. The nematode eggs are correctly identified even in complex, debris-filled images that are often difficult for experts to identify quickly. Our results illustrate the remarkable promise of applying deep learning approaches to phenotyping for pest assessment and management.

## Introduction

Disease and pathogen detection processes in plants are classified into direct and indirect methods^1^. Direct detection techniques, such as molecular markers using polymerase chain reactions (PCRs) and deoxyribonucleic acid (DNA) arrays, have been widely studied and analyzed. Lately, indirect disease detection techniques, via imaging for instance, have dramatically improved, and there is a significant interest in developing techniques for fast and automated analysis of such images^2^. In this context, object classification and detection in images become critical^2–5^. In certain cases, objects to be detected and enumerated by imaging techniques are much fewer or less conspicuous in the presence of extraneous objects^6^. Such cases have been termed *rare object detection problems*, with specific examples including pathogen detection (e.g., sexual and asexual spores)^7^, plant part detection^8^ and identification of foreign or unwanted objects from samples with applications in health, food and plant sciences and industries^1^. Our current study of identifying and quantifying soybean cyst nematode (SCN) eggs recovered from the soil using microscopic images is one such scenario where we demonstrate the effectiveness of our proposed deep learning paradigm in pathogen assessment.

The SCN is an obligate parasitic worm that develops from eggs to adults in as little as 24 days^9,10^. Feeding on soybean roots by the nematodes can inhibit the growth of the plants, cause yellowish discoloration of the leaves and increase the plants’ risk to more severe infections of diseases such as brown stem rot and sudden death syndrome^11^. SCN damage in the US alone is estimated to account, on an average, for a yield loss of about 3.5 million metric ton (one-third of the total loss)^12,13^, equivalent to >$1 billion per year in value^14^. The nematode had been managed successfully for decades by growing non-host crops, such as corn, in alternating years with soybean varieties that were bred to be resistant to the nematode. But virtually all of the resistant soybean varieties possessed the same set of resistance genes and, consequently, SCN populations have developed increased ability to reproduce on resistant soybean varieties^15^, leading to yield loss of the resistant varieties. Because of the loss of effectiveness of resistance, there is renewed need to monitor SCN population densities by collecting soil samples and quantifying the numbers of SCN eggs present in the soil.

**Figure 1:**
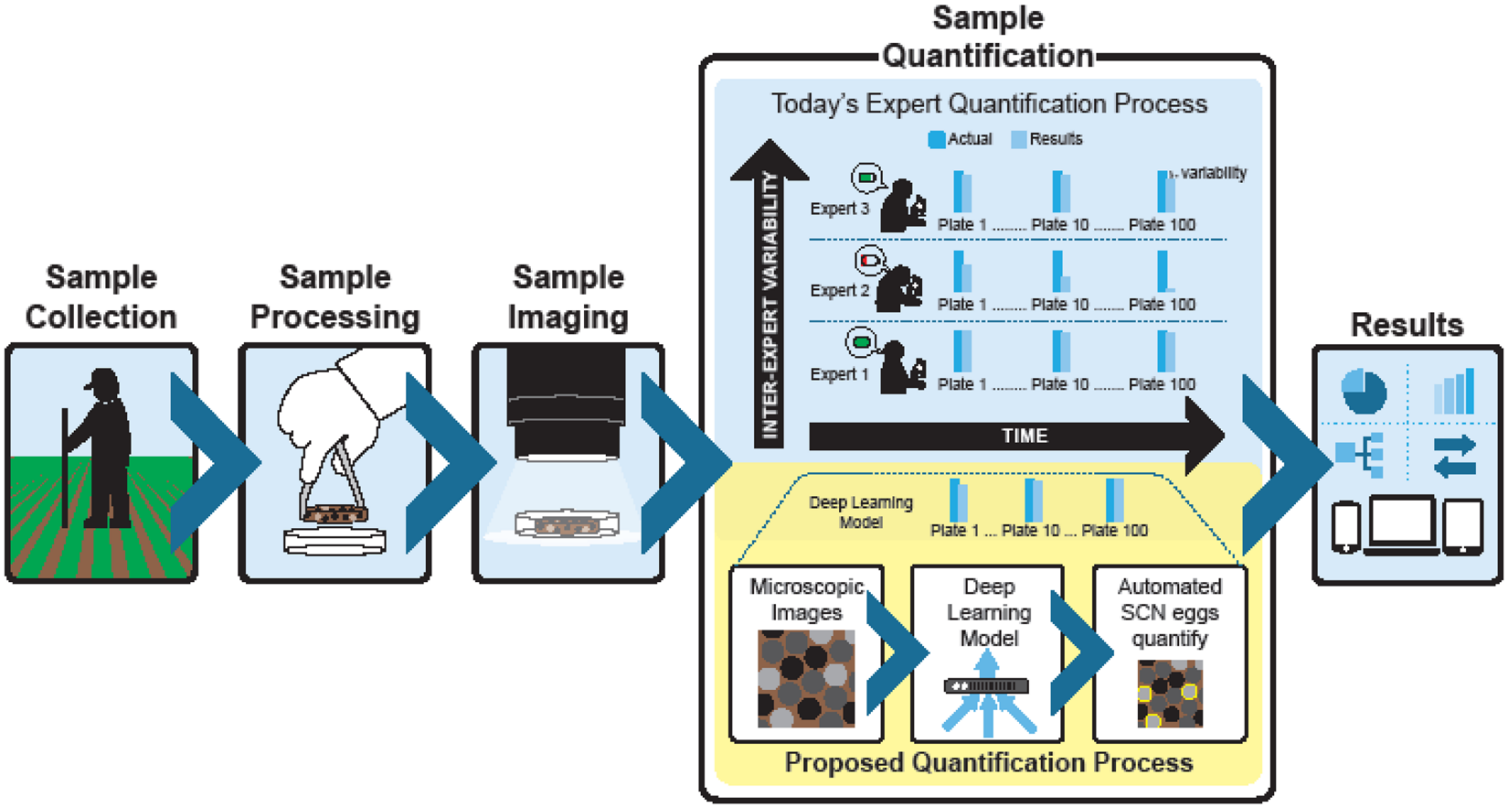
Approach overview showing the workflow that leads to the automated quantification process as an alternative to the human expert quantification which suffers from intra-rater and inter-rater variabilities.

After collection of soil samples from the field, SCN cysts (dead SCN females containing eggs) and debris are separated from other soil matter in processes known as elutriation^11^ or wet-sieving and decanting^16^. The resulting suspension of SCN cysts and debris is crushed by some means, commonly using a rubber stopper on the surface of a sieve^17^, then the eggs are recovered on a sieve, suspended in water with acid fuchsin and heated to color the eggs a pink-magenta color^18^ (*see* Supplementary Fig. 1). A sample of the stained egg suspension (typically 1 ml) is taken and placed on a nematode-counting slide that facilitates manual counting by trained technicians^19^ using an optical microscope (*see illustration in* Supplementary Fig. 3). While all other stages of the sampling and extraction process have undergone some degree of mechanization^20,21^, and can now be done in a fairly automated fashion, counting the number of SCN eggs has not yet been successfully automated, in spite of significant efforts using image processing strategies. Prior to 2015, most image-processing-based approaches have only been marginally successful^11^, exhibiting highly inconsistent performance and requiring frequent tuning, thus, limiting utility and ensuring that manual, human, visual identification and counting remains the gold standard. The difficulties in automation are primarily due to the resemblance of eggs with pervasive inorganic and organic debris, as well as the variability in coloration and occlusion of the eggs that occurs with eggs extracted from different soil types. The accurate counting of SCN eggs is further exacerbated by the occurrence of relatively few eggs per plate or image, hence ‘rare object detection’ problem (*see examples in* Supplementary Fig. 1 *to understand the complexity*). Even manual quantification of the SCN eggs on the frames require sufficient training because the debris can easily be confused with the SCN eggs and vice versa. Furthermore, trained technicians may have different levels of training and experience and get fatigued during this manual search process leading to both intra-rater and inter-rater variabilities (Fig. 1). Therefore, accurately recognizing and counting SCN eggs is a serious bottleneck to high-throughput assessment of SCN population densities as manual, human, visual counting is both *time consuming*, as well as *mistake*-*prone*.

**Figure 2:**
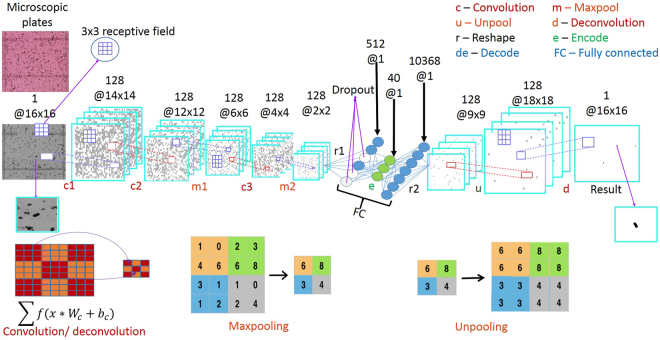
Deep Convolutional Selective Autoencoder architecture for rare object detection from images with application to SCN egg detection in cluttered microscopic images.

Deep learning approaches have recently had a transformative impact in image analytics, specifically in automatically learning complex patterns of interest for applications such as object recognition and scene understanding^5^, image de-noising and enhancement^22^, detection and labeling^23^. Within the deep learning paradigm, we design a novel end-to-end Convolutional Selective Autoencoder (CSAE)^24^ that possesses remarkable detection speed, consistency and accuracy in identifying SCN eggs across a wide variety of solution coloration, soil types and debris fields. The architecture was developed for this rare-object detection class of problems in such a manner that makes it robust to various data quality types without significant degradation in performance. A CSAE (illustrated in Fig. 2) has two main parts – the first part is called an encoder that learns to capture salient features from the input image in a layer-wise hierarchy while rejecting noise and other spurious disturbances, such that the patterns of interest in the input image can be faithfully reconstructed by the second part, called the decoder. The ‘selectivity’ aspect comes from the training procedure where the convolutional network model is trained in a way that it learns only to reconstruct an ‘egg’ pattern and mask/reject every other pattern in the input image. This supervised learning scheme uses a small set of microscopic images carefully annotated by experts to mark/highlight all SCN eggs to the best of their judgement.

We train the CSAE with labeled patches (image segments smaller than the entire image frame) from 644 microscopic image frames obtained from soil samples collected in fall 2015 and processed to extract the SCN eggs. Upon training, the model scans through the test images, patch by patch, and then identifies if each patch contains an SCN egg. Note that the detection of SCN egg(s) in each patch is affected by the presence of neighboring objects in the patch. This is particularly important for detection in the ‘high-cluttered’ images. We therefore fuse information from multiple overlapping local patches to provide a high level of confidence in estimating the existence of an SCN egg in a particular patch. While a high degree of overlap has the potential to improve the detection performance, it does require the processing of a higher number of patches. Therefore, the degree of overlap among the patches is determined to achieve an optimal trade-off between the detection accuracy and the computational complexity (i.e., detection time). Details of the data acquisition, labeling of the data, training of the model, and fusion of patch-level model inferences are given in the Methods section.

## Results and Discussion

**Figure 3:**
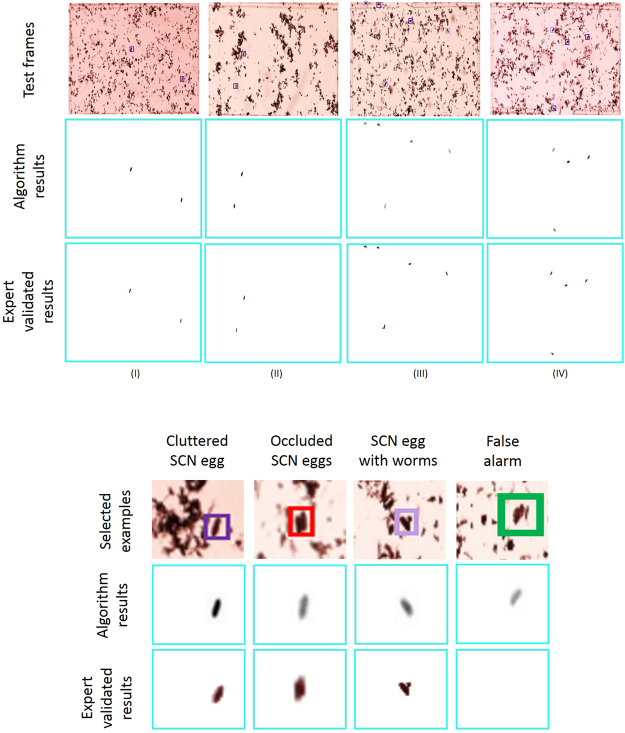
Sample detection results with images from diverse environment and different background staining for a: (I) set B1 example, (II) set B2 example, (III) set B13 example, and (IV) set B9 example; the dark purple boxes indicate highly confident detection, the light purple box indicates the low confidence detections, the red box indicates occluded eggs and the green box is a false alarm, while the low intensity in reconstruction suggests low detection confidence of the model.

Testing data sets were collected in spring 2016 from two different SCN-infested fields with different soil characteristics. The first farm, ‘A’, had 24 test sets (each test set consisting of 50 microscopic image frames) that were ‘less-cluttered’, while the second farm, ‘B’, had 24 test sets (each test set consisting of 50 microscopic image frames) that were ‘high-cluttered’. The sets which have more eggs and debris lumped together in different regions of the microscopic images are termed ‘high-cluttered’, while the ‘less-cluttered’ group contains sets with mostly distinct SCN eggs and debris (*For quantified definition of these image types*, *see Supplementary Section* - *Dataset and* Supplementary Fig. 2). Figure 3 illustrates the robustness of deep learning model in identifying SCN eggs under such widely varying conditions. Since the sizes of the objects of interest (i.e., eggs with area of roughly 5000 *μm*^2^) and the debris are much smaller compared to the entire image frames, we demonstrate the efficacy of the CSAE framework by zooming into a few representative areas of certain defined frames. The highlighted examples in Fig. 3 illustrates the model’s robustness to some extreme cases. The first example in Fig. 3(a) shows an egg in a significantly cluttered background. The CSAE model can reliably reconstruct the egg with high confidence (evidenced by the high pixel intensity value of the reconstructed egg object) while masking the other objects. The second example in Fig. 3(b) shows the ability of the machine learning model to detect and reconstruct an SCN egg object, where it is occluded in the input frame. In the third representative example shown in Fig. 3(c), an interesting phenomenon is shown that is observed in some cases where a worm is coming out of the egg. The CSAE model is able to reconstruct the egg in this scenario. Lastly, we show one of the very few false alarms obtained from the test frames in Fig. 3(d). In this case, while the object detected as an egg does have significant similarities in shape and size with an actual egg, the model associates a low pixel intensity signifying the low confidence it has in making this prediction. This suggests that CSAE identified objects can be automatically triage and selectively remove to further improve object recognition (*see Supplementary material*). Most interestingly, we had a few instances where the CSAE purportedly had false alarms, which were later confirmed to be actual eggs missed by the human expert.

**Figure 4:**
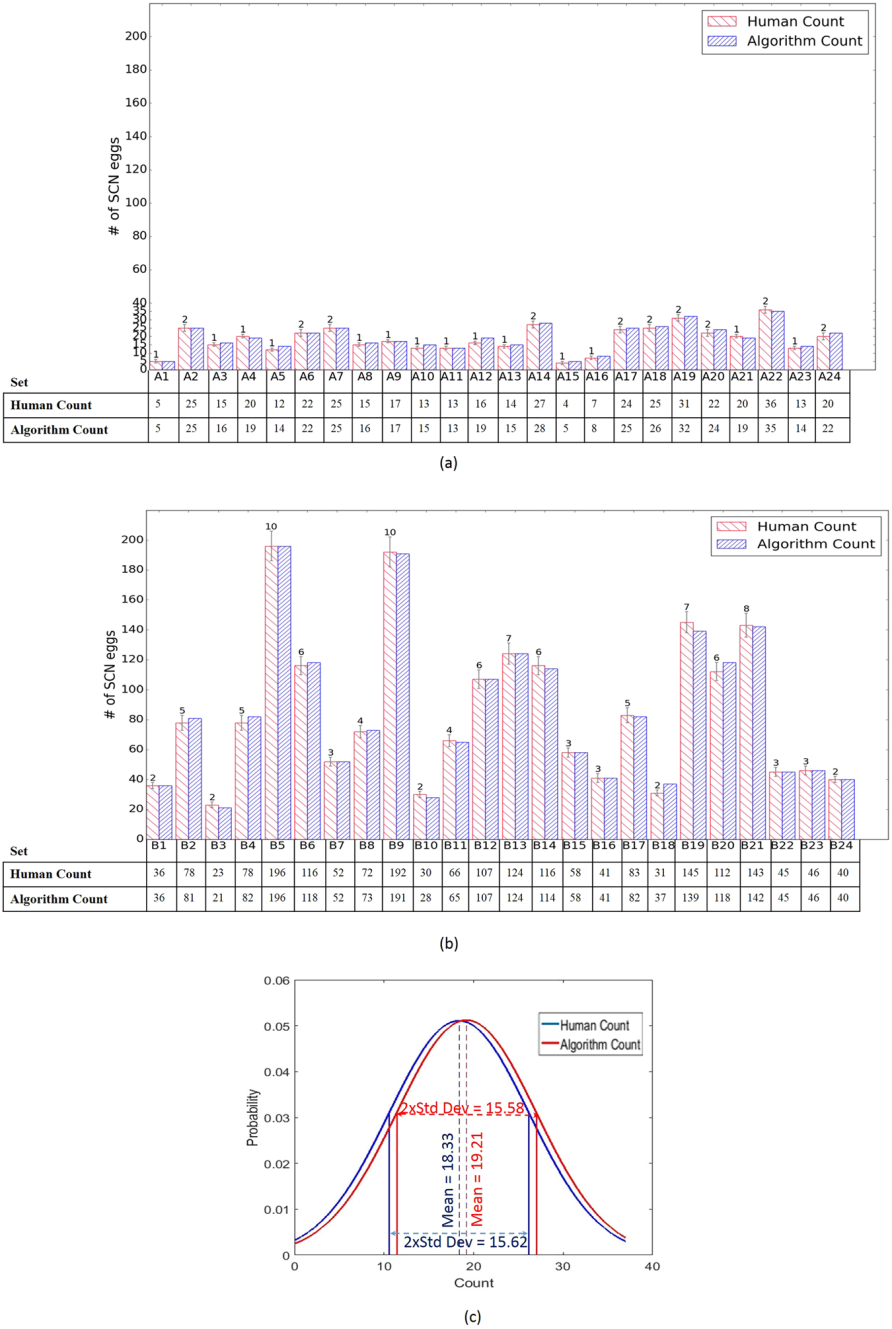
Statistics of transfer learning for spring 2016 image frames (total of 2400) testing results with model trained on fall 2015 (total of 644) frames for, (**a**) ‘less-cluttered’ group containing 24 sets labeled {A1, …, A24} of 50 frames, (**b**) ‘high-cluttered’ group containing 24 sets labeled {B1, …, B24} of 50 frames. The error bars are derived (as +/−5%) around the human counts for each of the image sets and (**c**) the distributions of the machine and human counts for the ‘high-cluttered’ and ‘less-cluttered’ results.

We provide the statistical summary of CSAE results in Fig. 4. First, we compare the machine counts on both ‘less-cluttered’ and ‘high-cluttered’ testing sets with human counts of SCN eggs. Note, each set count (human or machine) has 50 image frames. Error bars (+/−5%) are also included around the human counts for evaluating the machine counts. Egg counting performance is observed to be comparable with human experts for both the ‘less-cluttered’ and the ‘high-cluttered’ cases. While the ‘less-cluttered’ group in Fig. 4(a) have ≈92% (22 out of 24) of sets where detection accuracy is ≥95%, the ‘high-cluttered’ group in Fig. 4(b) have ≈96% (23 out of 24) of sets greater than or equal to 95% (*More result frames are included in the Supplementary material*). The ‘high-cluttered’ group contains more eggs alongside the debris and fewer misses hurt the detection less compared to the ‘less-cluttered’ group. We note however, that the ‘high-cluttered’ frames need a higher degree of overlap of patches (i.e., more computational time) to achieve the reported detection performance and the ‘less-cluttered’ frames have slightly less sensitivity to changes in the model learning hyper-parameters. However, more computational time issue is a mute argument with the availability of more powerful computers. A comparison of the distributions of the counts by human expert as well as machine is provided in Fig. 4(c). The distributions of both counts are seen to be close, judging by the mean values and the deviations around them. While the bar plots show overall egg count comparison for different test sets, we present detailed performance metrics in Table 1. The reported metrics are slight variants of the classical metrics such as false alarm and missed detection because it is infeasible to count the number of all objects in any frame as is required for the classical metric computation. Moreover, the few SCN eggs in each frame are already very laborious and time-consuming to count, therefore the debris, which are many more in number in the frames, will be rather unrealistic to count. The aggregated performance metrics show that our framework has significantly high degree of accuracy and robustness to noise and variability of the microscopic images. Furthermore, the imaging defects also include non-standard imaging processes (different image background lighting and orientations during the microscopic imaging), inconsistencies in intensity of colorization of eggs with the acid fuchsin stain, occlusion problems such as a non-egg blob on an egg, overlapping of SCN eggs and a worm (hatching juvenile) coming out of an egg.

**Table 1:** Performance metrics of algorithm on spring 2016 test sets, which are the ‘less-cluttered’ 24 sets labeled {A1, …, A24} having 50 frames, ‘high-cluttered’ group containing 24 sets labeled {B1, …, B24} of 50 frames and the aggregate performance of all the total 2400 testing images.

Adapted metric	Formulae	‘Less-cluttered’ group	‘High-cluttered’ group	Aggregate
Average detection accuracy	$\frac{\sum algorithm\,count}{\sum human\,count+error\,margin\,}$	$\frac{2036}{2150}=94.70 \%$	$\frac{459}{475}=97.00 \%$	$\frac{2495}{2625}=95.05 \%$
Average alarm-to-egg ratio	$\frac{\sum excess\,count\,(affected\,sets)}{\sum human\,count+error\,margin}$	$\frac{16}{2150}=0.75 \%$	$\frac{21}{475}=4.00 \%$	$\frac{37}{2625}=1.40 \%$
Average miss-to-egg ratio	$\frac{\sum undercounts\,(affected\,sets)}{\sum human\,count+error\,margin\,}$	$\frac{13}{2150}=0.60 \%$	$\frac{3}{475}=0.63 \%$	$\frac{16}{2625}=0.61 \%$
Average precision	$\frac{\sum algorithm\,count}{\sum human\,count+error\,margin+\sum excess\,count\,}$	$\frac{2036}{2166}=94.00$	$\frac{459}{496}=93.00 \%$	$\frac{2495}{2662}=93.73 \%$
F_1_- Score	$\frac{2\times Average\,precision\times Average\,detection\,accuracy}{Average\,precision+Average\,detection\,Accuracy}$	0.943	0.949	0.944

Error margin is found by taking 5% (i.e., the upper bound of the error bar) of the total human count for all image sets in each category.

Rapidly evolving algorithms in machine learning can lead to significant breakthroughs in complex plant phenotyping problems that have stubbornly resisted automation for decades^25^. Such approaches result in extremely consistent results that reliably relieve the expert of the monotony of dull repetitive tasks, while lowering quantification time and overall detection cost per sample.

In conclusion, a robust technique for identifying and counting rare objects in images have been proposed. The end-to-end convolutional selective autoencoder approach is trained for the current problem of searching for SCN eggs on microscopic images. A highly focused selectivity criterion is embedded in the training samples to aid CSAE discriminate the eggs from the highly similar debris. The current results show the robustness of the model, trained by the architecture, to identify the eggs despite the non-standardized lighting conditions, fuchsin coloring and clutter-level of the testing and training images. In general, CSAE reduces the cost of human counting as well as fatigue associated with it and therefore, can replace the manual practice of SCN egg counting. From a machine-driven SCN egg counting perspective, the current state-of-the-art, traditional computer vision technique using hand-crafted features^11^, claims to achieve a count within 10% of the expert counts over only few frames. In contrast, our CSAE model achieves an accuracy of around 96% and an overall F_1_-score of 0.944 on 2400 frames. CSAE is able to count 1440 frames/day/core (1 frame/minute) in very ‘high-cluttered’ frames, potentially reducing the number of days required for 108,000 frames (the number of frames considered in^11^) to only 70 days as opposed to 462 days as reported in^11^ using only a single core standard computer. However, in ‘less-cluttered’ frames the counting speed can increase dramatically to 242 frames/minute as we do not require significantly overlapping test patches from frames. With this speed, all 108,000 frames can be counted in <1 day. CSAE is also able to reduce cost of human counting as well as reduce fatigue associated with it. Importantly also, the method section will show how we reduce the cost of the extraction step by discarding the fuschin staining condition as we train the algorithm purely on grayscale images. Finally, we anticipate a proliferation of such ‘learning enhanced automation’ approaches that will have a transformative impact on agriculture, life and food sciences.

Some of the research directions that are currently being pursued are: (1) improving the object detection algorithm to handle occlusion of SCN eggs by debris as well as overlapping of multiple SCN eggs, (2) developing an easy interface such as a smart phone “app” for pathologists to easily estimate the SCN eggs on microscopic plates.

## Methods

### Soil sample and imaging

Dataset generation begins with collection of soil samples from SCN-infested fields by arbitrary placement of 25.4 mm diameter probes in a zigzag^11^ intervals on several farms in the state of Iowa of the United States. Soil samples were suspended in water, then sieved to separate root fragments and larger sized debris from the dead SCN females (called cysts) that are full of eggs^16^. The cysts recovered from the soil are physically crushed to break the cysts in order to release the SCN eggs held within^17^. Extracted SCN eggs and associated debris are placed in beakers with water and a few drops of acid fuchsin stain, heated to boiling, and then cooled^18^ when a 1 *cm*^3^ sample is placed in a nematode-counting slide. Images of the samples were taken under the microscope. To ensure that a wide variety of samples were used, two sets of soil samples collected from two fields each time were processed: the first was during fall 2015 and then the second, in the spring of 2016.

### SCN marking and curation

**Figure 5:**
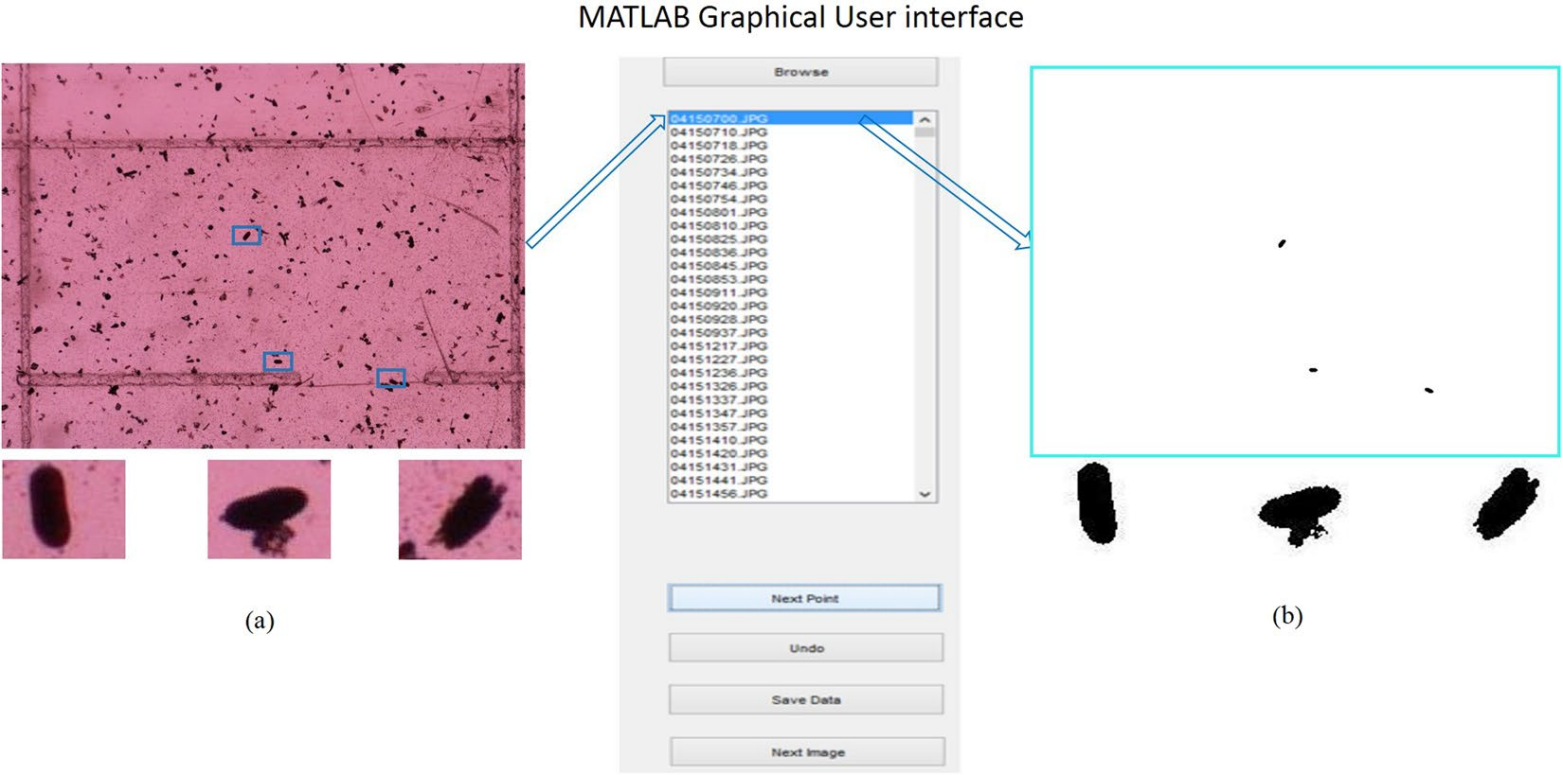
MATLAB GUI development of the mobile app that annotating the highlighted image of (**a**) based on expert information and producing the result in (**b**).

An easy-to-use marking tool was created to enable trained nematologists to label the eggs in the images. The tool was deployed on a touch-screen-enabled device as an app so that the location of the eggs can be marked. The location of every SCN egg is marked in each image and the pixel locations of each SCN egg stored into a database. The workflow of the mobile app, in its MATLAB based graphical user interface (GUI) development environment, is shown in Fig. 5. In the GUI, SCN eggs are the foreground objects, with the benefit of nematologists provided labels illustrated in Fig. 5(a), while all other non-egg objects are masked. The app shows in a sequential way, the set of microscopic frames opened while the highlighted one represents the annotated one.

Each image in the dataset was labeled, i.e., the location of every SCN egg in each image was precisely marked by two nematologists working independently. The use of two independent nematologists enable the quantification of intra- and inter-rater variability resulting from the deep learning approach. This process took more than two weeks of effort by each trained nematologist.

Although the app does a decent job of separating the foreground from the background as desired, it does not learn any model to automate further detection. The operating principle of the app however provided some ideas for learning to automate the detection and counting process. We have 644 annotated images and corresponding raw images constituted the training images. The images exhibited a wide diversity of soil samples, coloration intensities and SCN number densities. Additionally, spring 2016 images served as testing dataset. After testing was done, the images and results were returned to experts for validating the algorithm’s performance.

### Data preprocessing

The stained training images are converted back to gray-scale images (eliminating the requirement for fuchsin staining in the extraction stage) from all the training images. The pixels intensities of images, X and labels, Y pair, denoted by {(*X*^1^, *Y*^1^), …, (*X*^*I*^, *Y*^*I*^)} are normalized from [0, …, 255] → [0.0, …, 1.0]. This important pre-processing step is included to reduce the effects of random disturbances other than debris that may not be averagely uniform in either the test or training examples.

### Network training

The most intensive part of the analysis architecture was the training stage. The main idea in network training is to learn salient hierarchical features that are suitable for characterizing the SCNs^26–28^. Given ***I*** examples of M × N-dimension training images and labels that yield patches of sizes, m × n each, were randomly extracted from each of normalized training images *X*_*iMN*_ and at corresponding positions in the labels, *Y*_*iMN*_. The objects in the training patches are pose-centered in the selectivity function that is described later. At each convolution and deconvolution, a chosen (c × c) -size filter is convolved with a *z*_0_ –dimensional chosen mini-batches of stacked patches belonging to the training images. *z*_1_ –dimensional feature maps are derived with a rectified linear unit, ReLU^2^ activation, C, on the convolution that enforces local correlation. In the process, joint weights, *W*_*cc*_ and biases, *b*_*c*_ are expressed as,

1 $${\hat{Y}}_{{z}_{0}(m-c+1)(n-c+1)}^{j}=\sum C[{X}_{{z}_{0}mn}^{j}\ast {W}_{cc}^{j}+{b}_{c}^{j}]$$

where, $\hat{Y}$ is the algorithm’s estimate, * is a convolution operator, and j is the number of mini-batches present in the training examples. After some convolution layers, the feature maps are maxpooled^29^ to enhance invariance property of the convolved maps. These layers are alternated to produce the depth of the convolutional network, sometimes interlacing random unit dropout^30^ to control over fitting. This is especially useful to dropout units in the fully connected layer to reduce the number of over-fitted parameters.

The fully connected or bottleneck layers is the feature embedding layer. Here, the most important units explaining the output are encoded and decoded for each chosen mini-batch as,

2 $${\hat{Y}}_{e}^{j}=\sum E[{W}_{e}{\hat{Y}}_{R}^{j}+\,{b}_{e}^{j}]$$

3 $${\hat{Y}}_{d}^{j}=\sum D[{W}_{d}{\hat{Y}}_{e}^{j}+\,{b}_{d}^{j}]$$

where ${\hat{Y}}_{R}^{j}$ represents the vectorized inputs, ${\hat{Y}}_{e}^{j}$ and ${\hat{Y}}_{d}^{j}$ are the encoded and decoded outputs respectively, E and D are encoder and decoder functions which are also ReLUs. The set: {W, b} are weights and biases for the subscripted functions.

**Figure 6:**
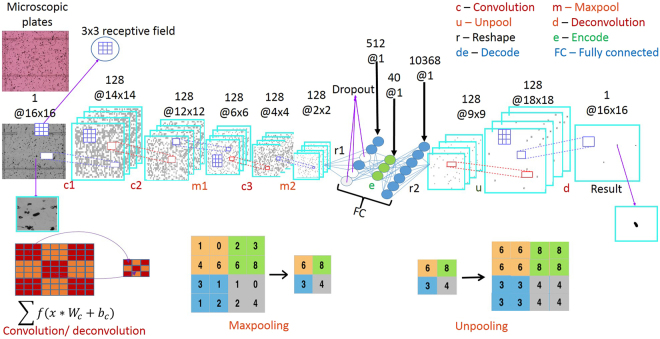
Convolutional selective autoencoder training architecture with a key describing the abbreviations in the architecture and image transformations implemented by the named core layers of the convolutional autoencoder.

An unpooling^31^ layer is added so that local neighbor’s features maps are again zoomed out by widening and stretching in a symmetric manner around the edge pixel based on the unpool. The output of the unpool layer is passed to a final deconvolution layer. The layer is similar to the convolution layer, and it helps to finally enforce local neighbors of the most effective feature mapping to the mini-batch label estimates. The estimates are then compared with the actual available training labels. Training for such comparison of the estimates and the labels is done in this case by optimizing an objective function formed by getting the error between the output estimate and the ground truth label of each local neighborhood. The optimization technique that is found most effective is based on the Nesterov momentum-based stochastic gradient descent method. The error in each case is back propagated to adjust the model parameters {W, b} in all the parameterize-able layers for a number of iteration called epochs. Such error plots are usually monitored and are a qualitative measure of the trainability of the algorithms. The model training was performed by reducing each labeled image into a set of non-overlapping patches with each patch either containing a single egg or no eggs. A total of labeled 1240360 image patches of (16 × 16) dimensions derived from images (M × N) = (480 × 640) that were collected in fall 2015 were used to train algorithm. A detailed breakdown of the total labeled image patches and their constituents is available (see Supplementary Table 1 for breakdown). The training architecture in Fig. 6 consists of convolution, maxpooling, reshape, fully connected, unpooling and deconvolution layers that are arranged in a hierarchical end-to-end manner. Unpooling and deconvolution layers facilitates architecture’s object recognition ability and confers the advantage of several more abstraction - layers^32^.

The architecture is an autoencoder^31^, because it has both the encoder and the decoder section. A review of the building blocks of such architectures can be found in literature^2,33^.

According to best practices in machine learning, training patches and corresponding labels are divided into 80% for training and 20% validating the algorithm, learning rate of 0.002 was improved by momentum of 0.975. Training is done on our graphics processing unit Titan Black machine having 2880 compute nodes and 6GB memory in Theano library (version 0.7)^34^, Lasagne (version 0.2) and Nolearn (version 0.1) wrappers of python. Filter sizes, (c × c) = (3 × 3) is found to be least costly in the mini-batches training where, z_0_ and z_1_ are each 128 patches. Training the model in Fig. 6 generates an overall of 743209 parameters (weights and biases).

### Selectivity function

In order to tackle the specific class of problems, namely rare object detection, which is complicated by the large level of similarity between the soil debris and other spurious background noise elements to the object of interest (i.e., SCN egg), we improve the architecture’s discriminative ability by the introduction of a *selectivity* function. Selectivity is an intermediate process between the pixel-wise and super pixel segmentation which learns to label as positive only the bounding box containing the object of interest. The algorithm enables the model to learn how to discard units that are undesirable based on the bounding boxes.

**Figure 7:**
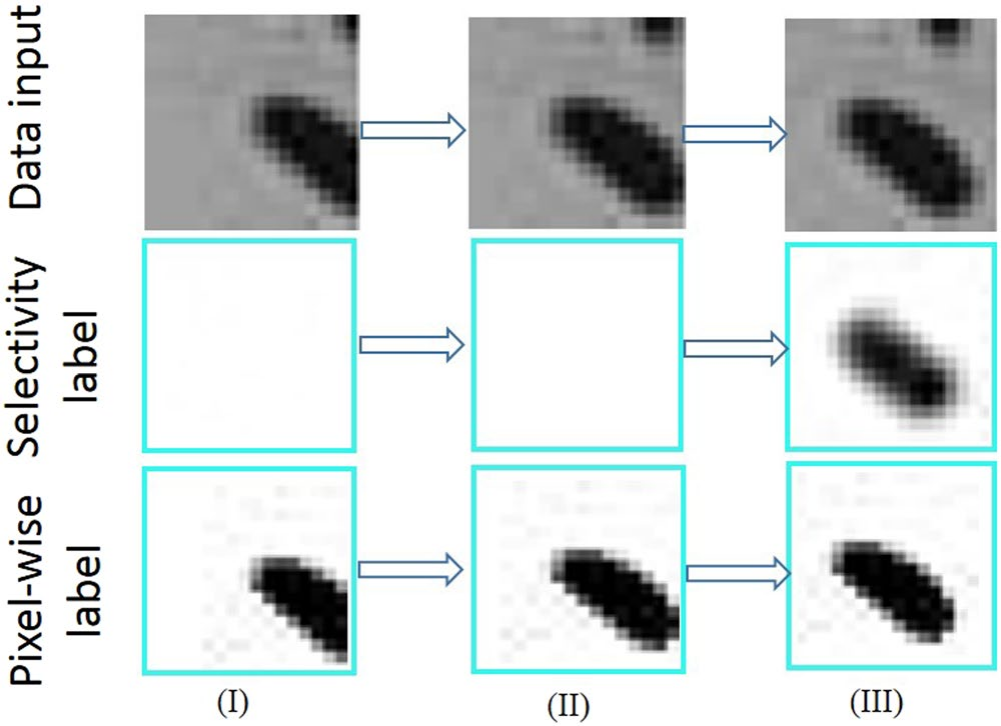
Patch-wise frames selected from video of the progression of an SCN egg example. Selectivity is seen to have superior properties than the pixel-wise semantic segmentation for solving similarity problem between the SCN eggs and debris.

The function only propagates the network activations/units that appear in patches that contain the fully visible SCN eggs, but rejects or masks activations/units that occur in patches where either the SCN eggs are not fully present or where there are no SCN eggs. Thus, apart from regular debris objects (that are visually quite different from eggs), the sets of units that are masked also include the debris that are visually similar to parts of eggs. Qualitatively, selectivity is a dynamic labeling function where the same bounding box can take on both an SCN egg at some point, while the image is masked as a debris at another point. A qualitative description of the function is captured in Fig. 7 and video-based illustration is provided in the attached *Supplementary Video*.

Although, selectivity is a dynamic labeling technique, it is the major ingredient that enabled the autoencoder network training to achieve the tasks. In Fig. 7, the SCN egg data input of either (I) or (II) are similar to debris in such a manner that a vanilla autoencoder network trained for semantic segmentation on pixel-wise basis would confuse the SCN eggs with debris and increase the likelihood of false alarms, but the selectivity ensures that the occurrence of such false positives is curtailed. We observe that the pose centering ability of the network is due to the selectivity criteria included. Selectivity also improves the SCN egg features learning ability of the autoencoder leading to a better accuracy.

### Network testing

At inference time, testing images were also patched to (m × n), similar to training images, but usually with local neighborhood overlap for effective fusion of local neighbor’s results. The number of patches for each test image is U × V.

4 $$U=\,\frac{M-m+{s}_{h}}{{s}_{h}},\,\,V=\frac{N-n+{s}_{w}}{{s}_{w}}$$

*s*_*h*_ and *s*_*w*_ are step sizes in the vertical and horizontal image dimensions. We stress here that the step sizes in Equation 4 mostly affect the computation complexity, which increases as the step size reduces (being inversely proportional for *s*_*h*_, *s*_*w*_ ${\epsilon }$ ***N***, where ***N*** is the set of natural numbers). This implies that reducing either *s*_*h*_ and *s*_*w*_ leads to a nonlinear increase in the number of patches to be independently analyzed^35^.

### Automated egg Counting

**Figure 8:**
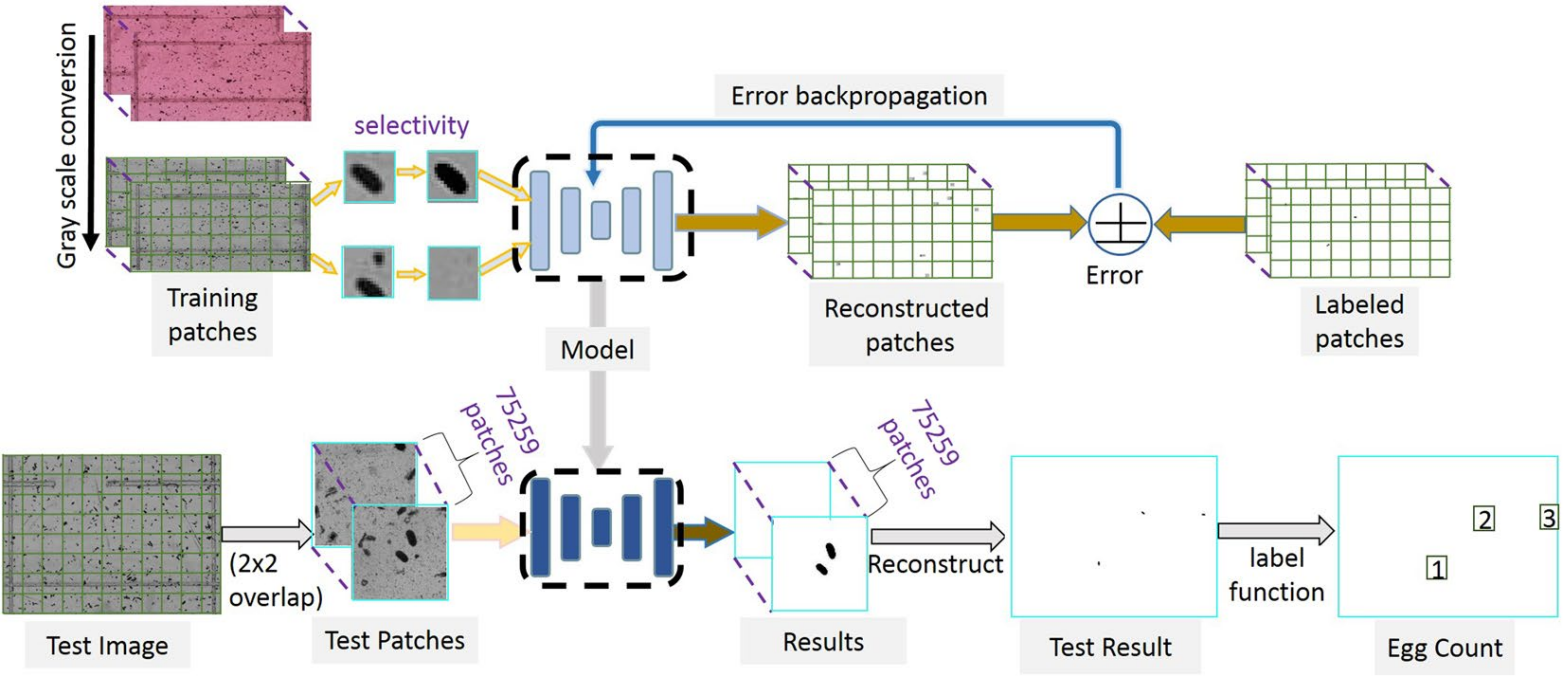
Algorithm implementation highlighting the workflow for training (at the top level) the network (dashed box) in forward activation and backpropagation and testing (at the bottom level) the trained network (dashed box) on an unseen test frame.

The detected eggs are then counted by using a matrix labeling function called *bwlabel*^36^. This simple image topology function is designed to count the number of connected components (blobs) that are activated in a binary conversion of an image. Its performance relies on the fundamental graph-theoretic definitions such as paths, components, neighbors, adjacency, among other terminologies. While there are deep learning networks which automates the counting of objects on frames^37^, the problem here uses a simple *bwlabel* function in order to ensure lower deployment overhead. The labeling algorithm begins with scanning along the vertices from the top-left node to the bottom-right node while assigning labels to the foreground pixels based on the labels of node of its adjacent neighbor. Then, equivalent labels are sorted into equivalent classes, after which different labels are assigned to the classes. Some more labels are then replaced with the labels assigned to the equivalent class in another scan through the image. The steps involved in the autoencoder implementation for the training and testing images are shown in Fig. 8. In all the 2400 frames, except 1 (test frame in Fig. 3 where multiple SCN eggs overlap), the counting tool was efficient, but an enhanced method is required for some rare occurrence of overlapping and/or occluded SCN eggs.

### Code and data availability

This protocol and source code, sample model, training and testing datasets are freely available for academic use in GitHub repository, https://github.com/pythonuser200/Convolutional_Selective_Autoencoder.

## Electronic supplementary material

Supplementary Information

Detection Video
