# Supplementary material for: A deep learning framework to discern and count microscopic nematode eggs
Source: Sci Rep. 2018 Jun 14;8:9145. doi: 10.1038/s41598-018-27272-w (PMC6002363; doi:10.1038/s41598-018-27272-w)
Supplement: Supplementary file 1 — Supplementary Information [file 41598_2018_27272_MOESM1_ESM.docx]

**A deep learning framework to discern and count microscopic nematode eggs**

**Adedotun Akintayo^1^, Gregory L. Tylka^2^, Asheesh K. Singh^3^, Baskar Ganapathysubramanian^1^, Arti Singh^3,+^, Soumik Sarkar^1,*^**

^1^Iowa State University, Mechanical Engineering Department, Ames, 50011, USA

^2^Iowa State University, Plant Pathology and Microbiology Department, Ames, 50011, USA

^3^Iowa State University, Agronomy Department, Ames, 50011, USA

^+^[arti@iastate.edu](mailto:arti@iastate.edu)

^[*](mailto:*soumiks@iastate.edu)^[soumiks@iastate.edu](mailto:*soumiks@iastate.edu)

**Supplementary material**

The soybean cyst nematode (SCN), *Heterodera glycines*, is the most damaging pathogen of soybean, *Glycine max*, in North America [1]. The nematodes can reduce yield by 30 - 50% or more. Yield losses increase as population densities (numbers) of the nematode increase in the soil. Current methods used to determine SCN egg population densities involve recovering the egg-filled, dead SCN females (called cysts) from the soil, extracting the eggs from the cysts, staining the eggs and then counting the eggs manually while viewing them through a microscope. Counting extracted SCN eggs must be done by trained personnel and can be difficult when the eggs are not sufficiently stained and/or when they are partially or mostly obstructed by debris in the microscope field of view. Depending on the texture of the soil from which the cysts and eggs were extracted, there can be much debris – both organic material and inorganic sediment particles.

The case study here is on automating the process of determining SCN population densities in soil by introducing a deep learning-based [2] [3] approach for automated detection and counting of the nematode eggs. Convolutional Selective Autoencoder (CSAE) [4] is employed to learn the rich descriptive features that discriminates the SCN eggs from soil particles and other debris. The network is automatically trained on expert-labeled [5] image plates - without expressly engineering features [6] about the objects of interest. The features-embedded model is deployed on newly provided images for blind evaluation.

Deep learning algorithms on the other hand have been implemented to blindly extract features from datasets, and match those features in a hierarchical layer-wise manner in deep networks [3], [7], [8], [9]. The architectures similar to those found in movie recommendation systems used in companies such as Netflix Inc. are another real-life application that has been facilitated by such learning procedures.

Application of the machine learning architecture to a task similar to ours [10] for automated phenotyping of embryos of the nematode *Caenorhabditis elegans* from videos taken under a microscope fitted with Nomarski differential interference contrast optics, by the addition of an energy-based model. In this work, we take a more recent approach by utilizing an end-to-end convolutional type networks [11], [12], called convolutional autoencoder which we train to be selective. The goal of a deep network is to learn the best representation of an objective from raw input with minimal input from the experts. The publicized benefits of artificial intelligent algorithms are tested to significantly automate SCN egg detection and counting. For the SCN egg quantification, a description of the background to the data collection and the stages involved in the algorithm helps to determine how to best preprocess the data.

**Dataset:** The dataset is made of microscopic image frames of SCN eggs obtained from soil samples consisting of 20 cm-deep soil cores collected using a 25.4 mm-diameter soil probe. Numerous soil samples were collected during fall 2015 from several areas (plots) on two separate farms having different SCN population densities at Iowa State University. The population density of SCN eggs was determined for a 100 cm^3^ subsample taken from each soil sample. The egg-filled, dead SCN females (called cysts) first were recovered from the soil [13], then the eggs were extracted from the cysts [14] and stained with acid fuchsin [15] in a suspension of tap water. A small sample of the extracted egg suspension, 1ml, is placed on a nematode-counting slide and images of the sample were taken using a camera through a microscope. Example images taken by the microscope are provided in Supplementary figure **1**.

About a thousand images were collected using this protocol. The images were then labeled by trained technicians. Labeling was accomplished by carefully examining each image and identifying and marking the location of every SCN egg present in that image.

The automation method is in the class of neural networks. Neural networks are examples of biologically inspired connectionist networks with units that are represented by the neurons, and are fired/activated by a mathematical combination of feature units of the data [16].

The first aim is to replace the current laborious method of human visual search for eggs in the microscopic by an automated machine that would ensure efficient counting of the SCN eggs. For that purpose, a machine learning method called convolutional selective autoencoder network has been optimized for the task of detection automation. The model learning is to be done with the proposed approach that portends significant capabilities in arriving at intelligent algorithms that: are able to reliably relieve human counters of stress of monotony; reduce the active engagement of man-in-the-loop; lowers the quantification time; lowers the detection cost per sample; and like every other software, promote re-use rather than re-training new specialists every time. Its re-use benefit is important because such models can be updated to capture newer - previously unseen - egg features for robustness.

*Supplementary figure 1: Microscopic image plate examples (I)-(IV) that were collected in fall 2015, expert-labeled and used for training the algorithm, and (V) and (VI) having different physical properties including the difficulty present in the former, are examples of frames collected in spring 2016 that are to be quantified by the automated tool. The purple boxes are the debris-similar, few SCN eggs (rare) present on the frames.*

Supplementary figure **1**(V) shows an examples of image from the ‘high-cluttered’ group while (VI) is an example from the ‘less-cluttered’ group. In this context, a way to distinguish ‘high-cluttered’ from ‘less-cluttered’ would be to consider how big the egg-debris clusters are in a frame. To compute this, we define a cluster in a frame to be a connected component with more than 50 foreground pixels (i.e., representing eggs and debris). Then we compare both the number of clusters (nc) and the average size of the clusters (sc) to distinguish between ‘high-cluttered’ (with high nc and sc) and ‘less-cluttered’ (with low nc and sc) frames. Supplementary figure **2** shows the outcome of such quantification process.


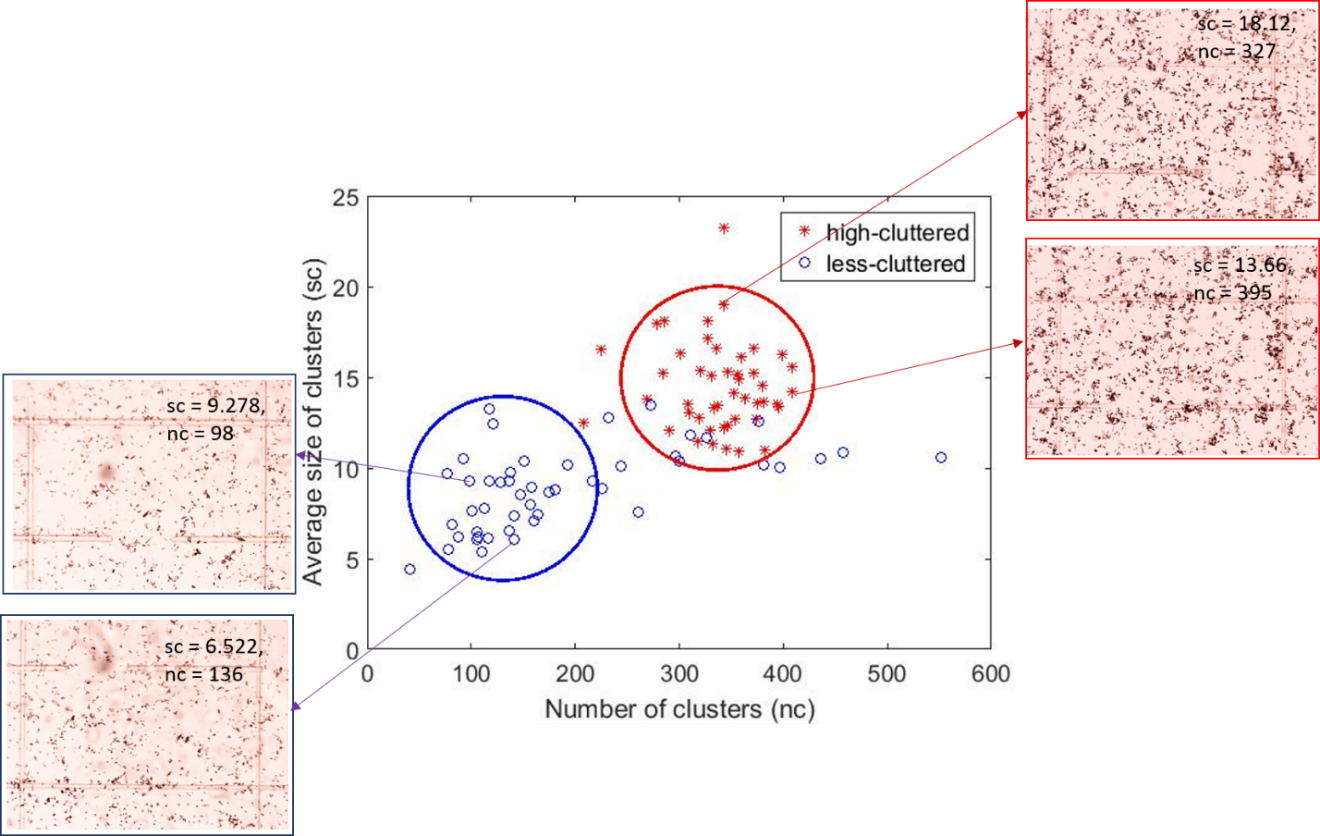


*Supplementary figure 2: showing the average cluster size against the number of clusters for the ‘less-cluttered’ and ‘high-cluttered’ frame sets. Two examples are highlighted for each group of frames.*

Expert versus Automatic detection and counting procedure:

Supplementary figure 3 provides a high level view of the current counting procedure as well as the proposed automated counting algorithm. The core of the automated improvement in detection and counting provided by the convolutional selective autoencoder, its requirements and important aspects of the underlying algorithm which are interesting to the machine learning community are described as follows.

*Supplementary figure 3: Automated improvement of current manual SCN detection and counting technique.*

**Algorithm Training:** The model learned by the algorithm is desired to be robust to various data quality types with only reasonably low degradation during detection for a reliable count and subsequently, inference. The rare object detection task of detection requires that the algorithm is developed to detect objects with low counts, and to discriminate them from other mostly physical feature-similar but unwanted objects. Increasingly many intelligence-requiring tasks that includes, but are not limited to: speech and text recognition [2], image denoising and enhancement [17], detection and labeling [4], [18] have benefited from the capability of similar architecture.

Available amount is limited because this stage requires the expert identifying and annotating the eggs. Both operations are usually part of the feature crafting that the automation seeks to eliminate. First, the algorithm needs to learn some cue from the human expert. Therefore, the minimum training dataset is derived for this purpose.

A summary of the breakdown of the extracted patches from the 1000 available expert-labeled example frames that are used to train the algorithm is provided on Supplementary table 1. The available datasets have been curated and augmented by cropping the eggs bounding box denoted by S, translating T and rotating R all the labeled L examples. Each example is a 480 x 640 – size image, and 16 x 16 local neighborhood boxes are derived from them.

*Supplementary table 1: Breakdown of training dataset, cropped – S, translated – T, rotated – R and labeled - L*

| Dataset type | Original dimension (16 x 16) | Final dimension |
| --- | --- | --- |
| S, T, R & L | 45432 x 10 | 454320 x 16 x 16 |
| S, R & L | 2524 x 10 | 25240 x 16 x 16 |
| L only | 634 x 480 x 640 | 760800 x 16 x 16 |
| Total |  | 1240360 x 16 x 16 |

The two main blocks in any of the algorithms is the forward activation of the units, followed by a backward training process, where the features are modified based on the goal. In the backward pass, several surrogate objective functions such as squared error function [19], cross-entropy function [20] hinge loss [21] which all have two class and multiple class varieties. It is the formulation of any of these loss functions that lead to changes in the features for the underlying data processes. Assume the feature set is modeled by $\theta=\{W,b\}$, and if the input X=x, and the target Y=y are random variables from an assumed distribution, than minimizing the cost function for $\theta\to\theta^{*}$ via,

|  | $\theta^{*}:=arg\min_{\theta}J\left( E_{\left( x,y \right)\sim P_{data}};\theta\right)$ | 1 |
| --- | --- | --- |

Where, J is the objective (loss) function. **E** is an expectation operator on samples (x, y) which are sampled$(\sim)$, from the data distribution $P_{data}$. Some tunable hyper-parameters can be considered for the optimization. They are the number of (X, Y) example pairs in the minibatch type optimization algorithm, although full dataset-target pair optimization, which eliminates this hyper-parameter, is always a first option. The second ensures that there is a scalable and faster multi-threading computation on a number of fewer pair providing the desired speed-up for a large dataset platform. Given the optimization algorithm, the optimum learning rate for the task also has to be determined. The known faults [22] with sub-optimal learning rates are the problems of not properly fitting the data to the model or being too greedily fast such that solutions end up becoming poorer. The objective functions are usually optimized by numerical algorithms such as the gradient descent algorithms [22], adaptive gradient (AdaGrad) [23], adaptive learning rate (AdaDelta) [11], adaptive moment (Adam) [24], root mean square propagation (RMSProp) [25] since the analytic optimization techniques would wrongly assume that only a single function is sufficient to parameterize the data.

Some other attempts at optimizing such functions have concentrated on regions around a known convex hull [31], [32]. In high dimensions [27], [26] propose that flat regions such as saddle points pose more threat than local minima. Hyper-parameters to be tuned in order to recover from the backlashes of such points are: random initialization e.g., [33] of the parameter value, addition of the patience hyper–parameter [34], regularization factor [35], dropout factor [36] and batch normalization [37]. An iterative parameter changes $\partial\theta_{k}$ similar to that explored for the Mixed National Institute of Science and Technology (MNIST) for digit recognition tasks [38] is implemented. It is given by,

|  | $\partial\theta_{k}=\mu^{k}\left( \partial\theta_{k-1} \right)-\left( 1-\mu^{k} \right)\epsilon^{k}\frac{1}{m}\sum_{m} \nabla_{\theta}\left[ J_{m}\left( \theta+\mu^{k}\left( \partial\theta_{k-1} \right) \right) \right]$ | 2 |
| --- | --- | --- |

Where $\mu^{k}$is the momentum hyper parameter (chosen to be Nesterov type), $\epsilon^{k}$is the learning rate, $\nabla_{\theta}\left[ J_{m}\left( \theta\right) \right]$ is the gradient of the cost function for each of the minibatch m at iteration step k. Given that change, the update of $\theta$ at the current step is found by,

|  | $\theta_{k}=\theta_{k-1}+\partial\theta_{k}$ | 3 |
| --- | --- | --- |

Supplementary equation 3 can be derived via the efficient back-propagation method [34]. The method is suitable for evaluating the feature changes at every layer of the algorithm such that it iteratively learns better features from either: more dataset or after more runs.

For the purpose of training the algorithm, a randomly selected portion (20% of total = 248072 x 16 x16) of the training dataset is usually held out to validate the results of the algorithm. The purpose of the validation set is to evaluate the performance of the algorithm on different in sample but unseen dataset for generalizability, which invariably helps to prevent overfitting. The remaining 80% is used to train the algorithm so that it iteratively minimizes some error function.

As described, the training problem deals with iteratively minimizing an objective function at each run through the forward and backpropagation stages. A progress of the error change with the resulting from the algorithm is shown in Supplementary figure **4**. The algorithm iteratively (in 100 runs) minimizes the errors for the training and validation datasets while learning better.

*Supplementary figure 4: Error history progress plot that resulted in the process of algorithm training.*

After the model is trained and validated to some satisfactory validation error level (usually,${10}^{-4}$), the learned model is implemented on the test data for evaluation of its results.

**Hardware support:** The results with architecture developed and trained with Nvidia® GeForce Titan X graphic cards that has 3072 CUDA cores, ensuring time-effective computation. The card has a 12GB of Virtual Random Access Memories (VRAMs), and a Compute Universal Device Architecture compute capability of 5.2 that ensures latest efficient drivers are utilized. Using multiple of such cards, there is large potentials for speed-up of computation by parallelization, thus ensuring that multiple farms can be examined within a short period, especially when specialized FPGAs are used.

**Supplementary results – detection images**

The results in Supplementary figure **5** are included as supplementary results of SCN egg detection produced by the algorithm. These image frames, although having a wide variety of non-uniform properties can be detected by the algorithm. Supplementary figure **5**(I) – (VIII) are the detection result for a randomly held out test set from the training frames of the samples collected and processed in the fall 2015. They show the large difference in background provided by the acid fuchsin staining compared with the testing frames of the spring 2016 samples. A feature crafted color dependent method would therefore fail to generalize on the new test frames, with the resultant effect being more human presence to supervise the quantification. Supplementary figure 5(IX) –XVI) are the detection results for the new test frames (spring 2016 sample) which themselves have variabilities that may affect feature crafting. Generally, selectivity training function helps to reduce false alarms which would have occurred as an effect of trying to increase the detection accuracy. On the average, the bulk of machine’s mistakes are missed detections, which were probably due to rare examples (in the training set), for example, worms on some eggs.

*Supplementary figure 5: Sample results of highly confident machine detection in dark purple boxes, low confidence machine detection in light purple, false alarms in green boxes, missed detections in yellow boxes and mislabeled eggs (as debris in the training set) in blue box for: (I) – (VIII), which are detection of 8 of 10 frames of fall 2015 held out from the training frames for blind testing and (IX) – (XVI), which are detection results from more of spring 2016 testing dataset that were unseen by the algorithm.*

**Supplementary results – detection video**

We show a detection snippet that demonstrates how a convolutional autoencoder that is trained to be selective detects the soybean cyst nematode eggs when the object is completely and rejects it when not fully seen - to avoid confusing an SCN egg with the highly similar debris particles on the frame. This was the power that helped reject many false positives and achieve state-of-the-art accuracy on the SCN egg detection task.

The video is also available in the attached video (Supplementary video) or on YouTube ( https://www.youtube.com/watch?v=p298CK1O6co).

# References

| [1] | T. W. Allen, C. A. Bradley, A. J. Sisson, E. Byamukama, M. I. Chilvers, C. M. Coker, A. A. Collins, J. P. Damicone, N. S. Dufault, P. D. Esker, T. R. Faske, L. J. Giesler, A. P. Grybauskas, D. E. Hershman, C. A. Hollier, T. Isakeit, D. J. Jardine, H. M. Kelly, R. C. Kumerait, N. M. Kleczewski, M. A. Newman, L. Osborne, C. Overstreet, G. B. Padgett, P. M. Phipps, P. P. Price, E. J. Sikora, D. L. Smith, T. N. Spurlock, C. A. Tande, A. U. Tenuta, K. A. Wise, J. A. Wrather, A. E. Dorrance, S. R. Koenning, J. E. Kurle, D. K. Malvick, S. G. Markell and H. L. Mehl, "Soybean Yield Loss Estimates Due to Diseases in the United States and Ontario, Canada, from 2010 to 2014," *The American Phytopathological Society,* vol. 18, no. 1, pp. 19-27, 2017. |
| --- | --- |
| [2] | Y. LeCun and Y. Bengio, "Convolutional networks for images, speech and time-series," in *The Handbook of brain Theory and Neural network*, MIT Press, 1998. |
| [3] | A. Krizhevsky, I. Sutskever and G. E. Hinton, "Imagenet classification with deep convolutional neural networks," *Neural Information Processing Systems,* p. 9, 2012. |
| [4] | A. Akintayo, K. G. Lore, S. Sarkar and S. Sarkar, "Prognostics of Combustion Instabilities from Hi-speed Flame Video using A Deep Convolutional Selective Autoencoder," *International Journal of Prognostics and Health Management,* vol. 7, no. 023 (Special Issue Big Data and Analytics), pp. 1-14, 2016. |
| [5] | Laboratory of Gregory L. Tylka, "Routine soil processing for SCN egg counts," Iowa State University, [Online]. Available: http://www.plantpath.iastate.edu/tylkalab/routine-soil-processing-scn-egg-counts. [Accessed 24 September 2016]. |
| [6] | F. A. Syed, *Development of an Automated System for Extraction and Quantification of Soybean Cyst Nematode (SCN) Eggs and Cysts,* Chicago: Illinois Digital Environment for Access to Learning and Scholarship (IDEAL), 2015. |
| [7] | G. Hinton and R. Salakhutdinov, "Reducing the dimensionality of data with neural," *Science,* pp. 504-507, 2006. |
| [8] | Y. Bengio, P. Lamblin, D. Popovici and H. and Larochelle, "Greedy layer-wise training of deep networks," *Advances in Neural Information Processing Systems,* 2007. |
| [9] | P. Vincent, H. Larochelle and Y. Bengio, "Extracting and composing robust features with denoising autoencoders," *Proceedings of the 25th International Conference on Machine Learning,* pp. 1096-1103, 2008. |
| [10] | F. Ning, D. Delhomme, Y. LeCun, f. Piano, L. Bottou and P. E. Barbano, "Toward Automatic Phenotyping of Developing Embryos from Videos," *IEEE Transactions on Image Processing,* vol. 14, no. 9, pp. 1360-1371, 2005. |
| [11] | M. D. Zeiler and B. Fergus, "Visualizing and Understanding Convolutional Networks," *ECCV,* vol. 8689, pp. 813-833, 2014. |
| [12] | J. Long, E. Shelhamer and T. Darrall, "Fully Convolutional Networks for semantic Segmentation," *Computer Vision and Pattern Recognition,* pp. 3431-3440, 2015. |
| [13] | J. W. Gerdemann, "Relation of a Large Soil-Borne Spore to Phycomycetous Mycorrhizal Infections," *Mycologia,* vol. 47, no. 5, pp. 619-632, 1955. |
| [14] | J. Faghihi and J. M. Feriss, "An Efficient New Device to Release eggs From Heterodera glycines," *Journal of Nematology,* vol. 32, no. 4, pp. 411-413, 2000. |
| [15] | T. L. Niblack, R. D. Heinz, G. S. Smith and P. A. Donald, "Density, Distribution, and Diversity of Heterodera glycines in Missouri," *Supplement to Journal of Nematology,* vol. 25, no. 4S, pp. 880-886, 1993. |
| [16] | M. B. Dalva, M. Weliky and L. C. Katz, "Relationships between local synaptic connections and orientation domains in primary visual cortex," *Science Direct,* pp. 871-880, 1997. |
| [17] | K. G. Lore, A. Akintayo and S. Sarkar, "LLNet: A deep autoencoder approach to natural low-light image enhancement," *Elsevier Journal of Pattern Recognition,* no. j_patcog2016.06.008, pp. 1-13, 2017. |
| [18] | C. Farabet, C. Couprie, L. Najman and Y. LeCun, "Learning hierarchical features for scene labeling," *exdb,* pp. 1-15, 2013. |
| [19] | R. Jafri and H. R. Arabnia, "A survey of face recognition techniques," *Journal of Information Processing systems,* pp. 42-68, 2009. |
| [20] | S. Kullback and R. Liebler, "On information sufficieny," *The annals of mathematical statistics,* pp. 1-10, 1951. |
| [21] | S. Chen and Y. Wang, "Convolutional neural networks and convex optimization," University of California, Department of Electrical and Computer Engineering., San Diego, 2013. |
| [22] | J. Martens, "Deep learning via Hessian-free optimization," *International Conference on Machine Learning,* pp. 1-8, 2010. |
| [23] | J. Duchi, E. Hazan and Y. Singer, "Adaptive subgradient methods for online learning," *Journal of Machine Learning Research,* pp. 2121-2159, 2011. |
| [24] | D. P. Kingma and J. L. Ba, "Adam: A method for stochastic optimization," *International conference on Learning and Recognition,* pp. 1-15, 2015. |
| [25] | Y. Dauphin, H. DeVries, J. Chung and Y. Bengio, "Rmsprop and equilibriated adaptive learning rate for non-convex optimization," 29 August 2015. [Online]. Available: https://arxiv.org/pdf/1502.04390.pdf. |
| [26] | E. J. Candes, X. Li and M. Soltanolkotabi, "Phase retrieval via wirtinger flow: Theory and algorithms," *IEEE Transactions on Informatiion Theory,* pp. 1985-2007, 2015. |
| [27] | G. Wang and G. B. Giannakis, "Solving random systems of quadratic equations via truncated generalized gradient flow," 16 October 2016. [Online]. Available: https://arxiv.org/pdf/1605.08285.pdf. |
| [28] | X. Glorot and Y. Bengio, *Journal of Machine Learning Research,* pp. 249-256, 2010. |
| [29] | Y. LeCun, B. Boser, J. S. Denker, D. Henderson, R. E. Howard, W. Hubbard and I. D. Jackel, "Handwritten digit recognition with a back-propagation network.," *exdb,* 1990. |
| [30] | C. M. Bishop, Pattern Recognition and Machine Learning, Cambridge: Springer Science-Business Media LLC, 2006. |
| [31] | N. Srivasta, G. Hinton, A. Krizhevsky, I. Sutskever and R. Salakhutdinov, "Dropout: A simple way to prevent neural networks from overfitting," *Journal of Machine Learning Research,* pp. 1929-1958, 2014. |
| [32] | S. Ioffe and C. Segzedy, "Batch normalization: Accelerating deep network training by reducing the internal covariate shift," 2 March 2015. [Online]. Available: https://arxiv.org/pdf/1502.03167.pdf. |
| [33] | I. Sutskever, J. Martens, G. Dahl and G. Hinton, "On the importance of initialization and momentum in deep learning," *International conference on Machine Learning,* 2013. |
| [34] | D. E. Rumelhart and J. L. McClelland, A framework for parallel distributed processing: Explorations in the microstrcuture cognition, Cambridge, MA, 1986. |
